# Remarks on Syphilitic Affections of the Eye

**Published:** 1882-05

**Authors:** L. De Wecker, Lucien Howe


### Translations.

### Remarks on Syphilitic Affections of the Eye—Extract from a Lecture by Prof. L. de Wecker.

From the French by Lucien Howe, M. D.

Let me complete this lecture by a few remarks concerning the manifestations of specific disease. It is next to impossible to give exact figures regarding the percentage of syphilitic affections, especially as some ocular troubles, which are probably due to that, are not manifested by any distinctive characteristics. It is fair to say, however, that counting all eye troubles together, those resulting from syphilis hardly exceed 15. per cent., thus, while the sword of Damocles hangs above the head of many an affected one, it only falls comparatively seldom.

As to the part of the eye most liable to be involved, that depends somewhat on the original trouble being one which is acquired or hereditary; in the former case, showing itself principally in the iris, in the latter, in the cornea. Next to the iris, the parts most liable to be involved in the acquired form, are the choroid, retina, optic nerve with its coverings, the sclerotic, cornea muscles and lids. In the hereditary form, next to the cornea, comes the iris and choroid, the walls of the orbit, and especially the lachrymal sac as most apt to become diseased.

Considering, for a moment, the ocular manifestations of the acquired form, we find them much more frequent in men than in women, and this, in the proportion of almost two to one, while there is surely no such inequality in the numbers of those having syphilis. Nor can this fact be explained by any increased difficulty in recognizing that disease among females, for all attempts at secrecy and reserve are usually laid aside, when the patient finds herself threatened with a serious affection of the eye, and naturally fearful as to the result.

Another fact worthy of notice is, that the ocular manifestations of the disease show a preference for rather advanced age, exhibiting themselves in persons from forty to fifty, or thirty to forty years of age, although the original infections are ordinarily during youth. It is therefore fair to infer that the tendency of syphilis to attack this organ, of our most valuable sense, is greater, in proportion as the individual is older.

Still a third point on which I would lay stress, is that the younger the subject, the less liable are the ocular symptoms to be accompanied by anatomical changes, which are diagnostic of specific disease. While on the contrary, when syphilis has taken hold of an older person, we see more frequently that there is a development in the morbid products in some form of the typical gumma.

We know that simple iritis in a young syphilitic subject does not differ from iritis in a person otherwise healthy, except that the former merges readily into a choroiditis or a retino-choroiditis. This point of differential diagnosis becomes more evident in proportion as the individual is older. Moreover, experience has taught me, that an irido-choroiditis with sclerosis of the vessels and pigmentary degeneration, does not always stop at that point, but that similar changes go on in the nervous centres, accompanied by symptoms of cerebral syphilis, thus invading the centre of an organ (the brain), of which, before, only a prolongation had been attacked. In general, then, the ocular manifestations of syphilis recently acquired by a young person, are much less dangerous and less obstinate to treatment than those which result when the disease is acquired rather late in life. In the latter case, the tendency to disorganization is more noticeable, and the persistence of the malady is made evident, not only by different tissues being successfully involved, but by the frequent relapses which are apt to occur.

Why is it that the eye has the unenviable privilege of playing such an important role among this class of affections? Can it be because of the abundance of its lymphatics or because of the great vascularity of the iris and choroid? These questions are still undecided; nor is it yet established, as some authors think (Manz, Schubert), that certain causes acting within or upon the eye, such for example as traction on the choroid in the development of posterior staphyloma—it is not proven, I say, that such influences pre-dispose that organ to the manifestations of syphilis. But even if we are ignorant of some points in the etiology, a study of the subject at least gives a clue as to the therapeutic rules which should guide the practitioner. These can be summed up in three words. They are to act with promptness, energetically and persistently. Mercurials combined with the iodide of potassium, or what is known as the mixed treatment, together with absence of light and complete rest in a room comfortably warmed, are absolutely necessary .In case there seems to be the least danger of unusually severe inflammation, it is well to pursue a plan like the following: Employ freely mercurial frictions, using 12 to 15 grammes (three to four drachms) at a time, and let a clyster also be prescribed which contains from 2 to 4 grammes (30 to 60 grains) of iodide of potassium.

By taking care to avoid the mercurial sore mouth, this routine may be continued about a month, and after an equal period of cessation, again resumed. It is well, however, to keep up almost continuously the use of the iodide, with perhaps occasionally a little pilocarpine subcutaneously. I have more faith in the employment of inunctions than in injections of mercurial peptones, which are disagreeable and often poorly borne by the patients. Finally, it is in every way desirable when a person has suffered from any syphilitic affection of the eye, that he pursue some systematic plan to thoroughly eradicate it, such as has been advised by Fournier and by Martineau. This consists in resuming the treatment at the end of one, two or three months, and supplementing it with sulphur baths. These stimulate into activity any cutaneous signs of the disease which may still remain latent, and not only cause the mercurial to be borne a longer time, but also serve to indicate, by the amount of reaction produced, whether or not the disease may be considered as thoroughly cured.—*Annales d'Oculistique.*

